# ALS-L1023 from *Melissa officinalis* Alleviates Liver Fibrosis in a Non-Alcoholic Fatty Liver Disease Model

**DOI:** 10.3390/life13010100

**Published:** 2022-12-29

**Authors:** Eun Jeoung Lee, Yun Kim, Ji Eun Kim, Eileen Laurel Yoon, Sung Ryol Lee, Dae Won Jun

**Affiliations:** 1 Department of Translational Medical Science, Hanyang University Graduate School of Biomedical Science and Engineering, Seoul 04763, Republic of Korea; 2 Hanyang Medicine-Engineering-Bio Collaborative & Comprehensive Center for Drug Development, Hanyang University, Seoul 04763, Republic of Korea; 3 College of Pharmacy, Daegu Catholic University, Gyeongsan 38430, Republic of Korea; 4 Department of Internal Medicine, Hanyang University School of Medicine, Seoul 04763, Republic of Korea; 5 Hanyang Institute of Bioscience and Biotechnology, Hanyang University, Seoul 04763, Republic of Korea; 6 Department of Surgery, Kangbuk Samsung Hospital, Sungkyunkwan University School of Medicine, Seoul 03181, Republic of Korea

**Keywords:** non-alcoholic fatty liver disease, non-alcoholic steatohepatitis, liver fibrosis, angiogenesis, lemon balm

## Abstract

ALS-L1023 is an ingredient extracted from *Melissa officinalis* L. (Labiatae; lemon balm), which is known as a natural medicine that suppresses angiogenesis. Herein, we aimed to determine whether ALS-L1023 could alleviate liver fibrosis in the non-alcoholic fatty liver disease (NAFLD) model. C57BL/6 wild-type male mice (age, 6 weeks old) were fed a choline-deficient high-fat diet (CDHFD) for 10 weeks to induce NAFLD. For the next 10 weeks, two groups of mice received the test drug along with CDHFD. Two doses (a low dose, 800 mg/kg/day; and a high dose, 1200 mg/kg/day) of ALS-L1023 were selected and mixed with feed for administration. Obeticholic acid (OCA; 10 mg/kg/day) was used as the positive control. Biochemical analysis revealed that the ALS-L1023 low-dose group had significantly decreased alanine transaminase and aspartate transaminase. The area of fibrosis significantly decreased due to the administration of ALS-L1023, and the anti-fibrotic effect of ALS-L1023 was greater than that of OCA. RNA sequencing revealed that the responder group had lower expression of genes related to the hedgehog-signaling pathway than the non-responder group. ALS-L1023 may exert anti-fibrotic effects in the NAFLD model, suggesting that it may provide potential benefits for the treatment of liver fibrosis.

## 1. Introduction

Non-alcoholic fatty liver disease (NAFLD) is one of the most common liver diseases worldwide, with a prevalence of approximately 25% [1]. NAFLD encompasses a variety of liver abnormalities, including non-alcoholic fatty liver (NAFL) or simple hepatic steatosis, and non-alcoholic steatohepatitis (NASH), a more serious condition associated with inflammation in and damage to the hepatocytes, which is generally accompanied by liver fibrosis. NASH can also progress to cirrhosis and hepatocellular carcinoma [2]. The prevalence of NASH is expected to increase to 56% over the next 10 years [3]. Chronic liver injury, such as NASH, hepatitis B and/or hepatitis C infections, and alcohol cause liver fibrosis by activating myofibroblasts in the liver, which secrete extracellular matrix proteins that form fibrous scars [4]. Myofibroblasts are the major source of extracellular matrix in liver fibrosis and activated hepatic stellate cells are considered to be one of the main sources of myofibroblasts [4]. Liver fibrosis is a factor that develops into the progressed phase of NAFLD [5]; however, there is no effective therapeutic option for liver fibrosis with liver transplantation as the only curative treatment in advanced disease stages [6].

In various organs during the growth and repair of damaged tissues, angiogenesis, the production of new capillaries from preexisting vasculature, is an active, growth factor-dependent, and hypoxia-induced phenomenon [7]. Although angiogenesis is essential for tissue growth and regeneration, accumulating research showed that it occurs in numerous organs during a variety of pathologic conditions. Data on hepatic angiogenesis in NAFLD animal models and humans imply that this phenomenon plays a role in the pathogenesis of NAFLD [8]. Angiogenesis occurs in chronic liver disorders as well, and it is associated with the advancement of liver fibrosis, regardless of the origin of the liver disease [9,10]. Diverse factors, including hypoxia, inflammation, and endothelial dysfunction, induce angiogenesis, which has the primary consequence of aggravating liver fibrosis and leading to cirrhosis [11]. Angiogenesis causes quantitative and qualitative changes in liver vessels, including the formation of new vessels and vascular remodeling [12]. The loss of fenestrae in the hepatic sinusoids as a result of gradual dedifferentiation in liver sinusoidal endothelial cells is a first sign of the fibrotic process [12].

ALS-L1023 is an ingredient extracted from *Melissa officinalis* L. (Labiatae; lemon balm), which grows wild in Europe and the Mediterranean. This lemon balm extract is also known as a natural medicine that suppresses angiogenesis [13]. The herbal composition, Ob-X, which includes the extract from *M. officinalis* was found to exert anti-angiogenic effects and reduce adipose tissue mass in obese mice [14]. The lemon-balm extract, ALS-L1023, was also found to alleviate fatty liver in high-fat diet (HFD)-induced NAFLD by reducing the production of visceral adipose tissue in mice, thereby supporting the notion that angiogenesis inhibitors could prevent obesity-induced human NAFLD [15]. ALS-L1023 also decreased the number of CD68^+^ macrophages and inflammatory cytokines in the adipose tissue of HFD mice [15]. Further, this extract reduced the inflammation in diet-induced obesity and NAFLD in female ovariectomized mice and relieved oxidative stress through Akt activation [16]. These results suggest that angiogenesis inhibitors may be an effective therapeutic option for obesity and NAFLD. The resolution of NASH without worsening of fibrosis or one or more stages of reduction in fibrosis without worsening NASH is now accepted as a surrogate endpoint of NASH clinical trials. Anti-fibrotic effects are essential properties of NASH drugs. Pathologic angiogenesis is intimately associated with the fibrogenic progression of chronic liver diseases as angiogenesis plays a critical role in any wound healing response, with wound healing as a typical mechanism involved in the progression of chronic liver disease [17]. However, whether ALS-L1023 can reduce intrahepatic fibrosis in NAFLD remains unknown.

As described in previous studies, ALS-L1023 caused a decrease in lipid synthesis and inflammation. Currently, ALS-L1023 is in the early phase IIa clinical trial for patients with NAFLD. However, the anti-fibrotic effects of ALS-L1023 are still under evaluation, and knowledge of the relevant mechanism of action remains limited. Therefore, this study aimed to determine whether ALS-L1023 could alleviate liver fibrosis in the NAFLD model. Further, exploratory research was conducted on the signaling pathway using mRNA sequencing.

## 2. Materials and Methods

### 2.1. Preparation of ALS-L1023

ALS-L1023 was manufactured via activity-guided fractionation from *M. officinalis* leaves purchased from Alfred Galke GmbH (Harz, Germany). Briefly, the dried *M. officinalis* leaves were subjected to extraction with aqueous ethanol, and the resulting extract was filtered and concentrated. The concentrated ethanol extract was further fractionated with ethyl acetate, concentrated, and dried to obtain ALS-L1023 in a dried powder form. ALS-L1023 was standardized with two reference compounds, rosmarinic acid and caffeic acid, by high-performance liquid chromatography [18]. On the Hitachi LC organizer, HPLC analysis was performed. The column had a thermostated temperature of 30 °C and was a Vydac protein and peptide C18 reversed-phase column (5 μm, 250 × 4.6 mm^2^). Mobile phases A and B were made up of 5% formic acid in water and methanol, respectively. The sample injection volume was 10 μL, the flow rate was 1.0 mL/min, and UV detection was conducted at 285 nm. Rosmarinic acid and caffeic acid had retention times of 7.33 and 4.18 min, respectively.

### 2.2. Animal Studies

**Figure 1:**
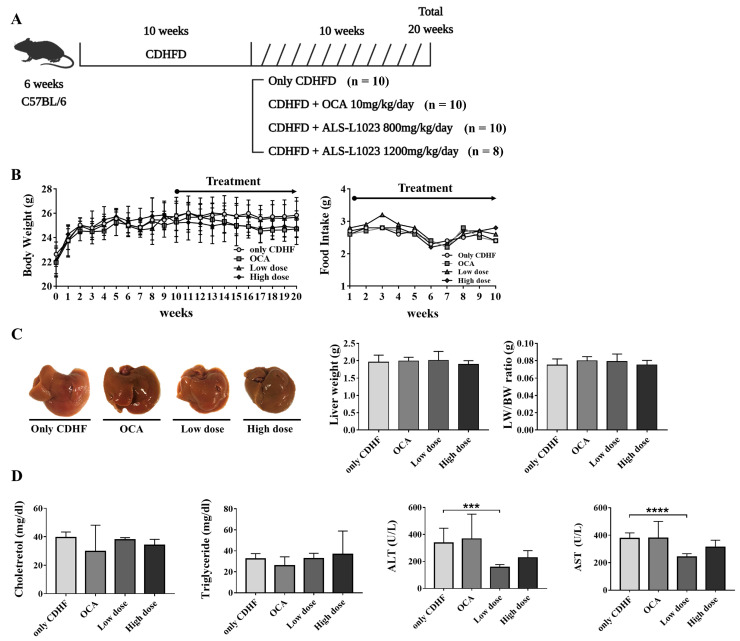
Animal study and the effect of ALS-L1023 on body weight, food intake, liver, and biochemical markers in the choline-deficient high-fat diet (CDHFD)-induced non-alcoholic fatty liver disease model. (**A**) Schematic of the animal study. (**B**) Body weight and food intake. (**C**) Liver images of the CDHF, obeticholic acid (OCA), ALS-L1023 low dose, and ALS-L1023 high dose groups. (**D**) Biochemical marker analysis. Data are presented as mean ± standard deviation and were analyzed by Mann-Whitney U test. *** *p* < 0.001, **** *p* < 0.0001 compared to the CDHF group.

C57BL/6 wild-type male mice (age, 6 weeks old) were purchased from Dae Han Bio Link (Eumseoung, Republic of Korea), bred, and managed at the Hanyang Laboratory Animal Research Center (Seoul, Republic of Korea). The animals were housed in a facility free of specific pathogens under controlled temperature (23 °C ± 2 °C) and humidity (55 ± 5%) conditions, with a 12 h artificial light/dark cycle. All animals (*n* = 38) were fed a choline-deficient high-fat diet (CDHFD [L-Amino acid diet with 62% kcal fat, 0.1% methionine and no added choline, 18% kcal protein, and 21% kcal carbohydrate]; Research Diets, Inc., New Brunswick, NJ, USA) for 10 weeks to induce NAFLD (Figure 1A). For the next 10 weeks, two groups of mice received the test drug along with CDHFD. Two doses of the lemon balm extract, ALS-L1023, were tested in each group (low dose (*n* = 10), 800 mg/kg/day and high dose (*n* = 8), 1200 mg/kg/day) and mixed with feed for administration. The control group (*n* = 10) was fed with only CDHFD. Obeticholic acid (OCA; 10 mg/kg/day) was used as the positive control (*n* = 10). Randomization was performed so that there was not much difference in body weight by group. The rationale of sample size determination was based on experience from previous animal studies and was not based on power calculations. Body weight was measured weekly until the end of the study, and food intake was measured weekly after treatment. Liver and blood samples were obtained at week 20, and the liver weight was measured. Anesthesia was performed by intraperitoneal injection of a general anesthetic mixed with Zoletil and Rompun to reduce pain, suffering and distress. The study was approved by the Hanyang University Institutional Animal Care and Use Committee (2019-0114A), and the research protocol was prepared before the study.

### 2.3. Biochemical Analysis

For biochemical analysis, the euthanized mice were subjected to thoracotomy. The blood samples were collected through cardiac puncture using an insulin syringe and serum was obtained by centrifuging the blood (3000 rpm, 4 °C, 15 min). The isolated sera were stored at −80 °C until analysis. Total cholesterol, triglyceride, alanine transaminase (ALT), and aspartate transaminase (AST) were measured using a Hitachi 747 autoanalyzer (Hitachi, Tokyo, Japan) according to the manufacturer’s instructions (Knotus Co. Ltd., Guri, Republic of Korea).

### 2.4. Histological Analysis

Liver tissue was fixed using a tissue fixation solution (4% paraformaldehyde) and embedded in paraffin. Paraffin blocks were sectioned into 4-μm slices and subjected to hematoxylin and eosin (H&E) staining and Sirius red staining. The stained sections were photographed using a slide scanner (AxioSan.Z1; Zeiss, Oderkochen, Germany) and a light microscope (Leica DM 400 B; Leica, Wetzlar, Germany). The extent of steatosis, inflammation, and hepatocyte ballooning was scored according to the NAFLD Activity Score (NAS) system. Fibrosis was observed using a light microscope after staining with Sirius red, and the area of fibrosis (%) was measured using ImageJ by acquiring three images for each appropriate site of the liver per mouse. As the primary outcome measure, all histological data were assessed by an independent blinded researcher.

### 2.5. RNA Extraction and Quantitative Real-Time Polymerase Chain Reaction (qRT-PCR)

Total RNA was isolated from liver tissues using TRIzol reagent (Invitrogen Co., Carlsbad, CA, USA). RNA concentration was measured using a Nanodrop ND-2000 spectrophotometer (Thermo Scientific Inc., Waltham, MA, USA). The extracted RNA (3 μg) was synthesized into cDNA using reverse transcription (PrimeScript™ RT Reagent Kit; TaKaRa; Otsu, Shiga, Japan). qRT-PCR amplification was performed using a LightCycler 480 (Roche Diagnostics, Mannheim, Germany) with LightCycler 480 SYBR Green I Master mix (Roche Diagnostics, Mannheim, Germany). The crossing point of each sample was calculated using the LightCycler^®^ program. All tests were performed in triplicate. The measured values were normalized to those for GAPDH and β-actin. The sequences of all primers are described in Table 1.

### 2.6. RNA Isolation for RNA Sequencing

Total RNA was isolated using TRIzol reagent (Invitrogen, Waltham, MA, USA). RNA quality was assessed using an Agilent 2100 bioanalyzer (Agilent Technologies, Amstelveen, The Netherlands), and RNA quantification was performed using an ND-200 spectrophotometer (Thermo Inc., Waltham, MA, USA).

### 2.7. Library Preparation and RNA Sequencing

RNA sequencing was performed for the CDHFD (*n* = 3), ALS-L1023 responder (*n* = 3), and ALS-L1023 non-responder (*n* = 3) groups from the more effective dose group. A group that showed improvement in fibrosis (depending on the average of the area of fibrosis) was defined as a responder group whereas a group that did not show improvement in fibrosis was defined as a non-responder group. Libraries were prepared from total RNA using the NEBNext Ultra II Directional RNA-Seq Kit (NEW ENGLAND BioLabs, Inc., UK). mRNA was isolated using the Poly(A) RNA Selection Kit (LEXOGEN Inc., Vienna, Austria). The isolated mRNAs were used for cDNA synthesis and shearing, according to the manufacturer’s instructions. Indexing was performed using Illumina indexes 1–12. The enrichment step was performed using PCR. Libraries were subsequently checked using the Agilent 2100 Bioanalyzer (DNA High Sensitivity Kit) to determine the mean fragment size. Quantification was performed using a library quantification kit and a StepOne Real-Time PCR System (Life Technologies Inc., Carlsbad, CA, USA). High-throughput sequencing was performed as paired-end 100 sequencing using a NovaSeq 6000 (Illumina, Inc., San Diego, CA, USA).

### 2.8. RNA Sequencing Data Analysis

Quality control of the raw sequencing data was performed using FastQC [19]. Adapter and low-quality reads (<Q20) were removed using FASTX_Trimmer [20] and BBMap [21]. Thereafter, the trimmed reads were mapped to the reference genome using TopHat [22]. Gene expression levels were estimated using fragments per kilobase per million reads (FPKM) values by Cufflinks [23]. The FPKM values were normalized based on the quantile normalization method using EdgeR within R (R Development Core Team, 2016). Data mining and graphic visualization were performed using ExDEGA (Ebiogen Inc., Seoul, Republic of Korea). The expression profiles are used to do additional analysis such as differentially expressed genes. On the basis of the Kyoto Encyclopedia of Genes and Genomes (KEGG) pathway (http://www.genome.jp/kegg/pathway.html; accessed on 23 September 2022), gene enrichment and functional annotation analysis, as well as pathway analysis for a significant gene list, were performed. To identify potential function of the genes, Gene Set Enrichment Analysis (GSEA; http://software.broadinstitute.org/gsea/index.jsp) was conducted to detect whether a priori defined biological processes show statistically significant.

### 2.9. Western Blots

Liver tissue proteins were extracted using RIPA lysis buffer (GenDEPOT, Hanam, Republic of Korea). The extracted proteins (11 μg) were then loaded onto 10% sodium dodecyl sulfate-polyacrylamide gel for electrophoresis. The separated proteins were transferred onto nitrocellulose (NC) membranes (0.45 μm-pore size; Bio-Rad, Hercules, CA, USA), blocked with 1× EzBlock Chemi solution (ATTO, Tokyo, Japan) for 30 min, and incubated with the primary antibodies, α-SMA (1:1000; ab5694, Abcam, Cambridge, UK) and GAPDH (1:1000; GTX100118, Genetex, CA, USA), overnight at 4 °C. Membranes were then incubated with a secondary HRP-conjugated anti-rabbit antibody (1:5000; SA200-500, Jackson Immunoresearch, West Grove, PA, USA) for 1 h at 25 ± 2 °C. GAPDH was used as a protein loading control. Positive protein bands were visualized using the Dyne ECL STAR Western blotting Detection Kit (Dyne Bio, Seongnam, Republic of Korea), and the results were quantified using an image analyzer (Image Lab 3.0, Bio-Rad, Hercules, CA, USA).

### 2.10. Statistical Analysis

Statistical Package for GraphPad Prism 5.0 (GraphPad Software, Inc., San Diego, CA, USA) was used for statistical analyses. All data are expressed as the mean ± standard deviation (SD). Data were analyzed using the Mann-Whitney U test (if the general assumptions were met, e.g., not normally distributed) and one-way ANOVA (for multiple comparisons), and post hoc multiple comparisons were made with Tukey’s test (equal variances assumed). Statistical significance was set at *p* < 0.05.

## 3. Results

### 3.1. ALS-L1023 Decreased ALT and AST in the CDHFD Model

All animals were killed at 20 weeks; however, no significant difference was found between groups in mean final body weight (CDHF group: 25.8 ± 1.1 g, OCA group: 24.7 ± 0.7 g, ALS-L1023 low dose group: 25.7 ± 1.5 g, ALS-L1023 high dose group: 24.8 ± 1.3 g) and food intake (Figure 1B). Further, there was no significant difference in liver weight and liver weight/body weight ratio between the groups (Figure 1C). Based on the biochemical analysis, the ALS-L1023 low-dose group had significantly decreased ALT (157.0 ± 19.2 vs. 337.1 ± 94.8; *p* = 0.0004) and AST (241.2 ± 23.1 vs. 376.2 ± 35.4; *p* < 0.0001) levels compared to the CDHFD group, despite a lack of significant difference in total cholesterol and triglyceride among all groups (Figure 1D).

### 3.2. ALS-L1023 Decreased the Area of Fibrosis in the CDHFD Model

**Figure 2:**
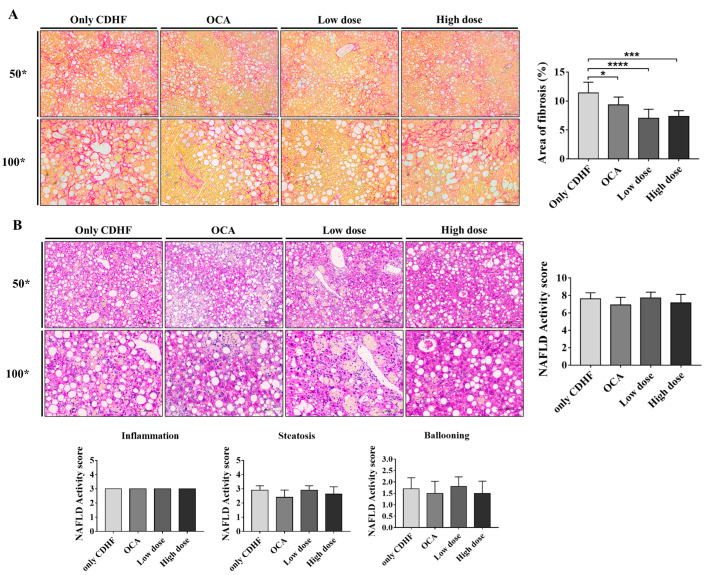
Effect of ALS-L1023 on fibrosis and Non-alcoholic fatty liver disease (NAFLD) Activity score in the liver. (**A**) Liver Sirius stain and area of fibrosis (%), 50×, 100×. (**B**) Liver H&E stain and NAFLD Activity score, 50×, 100×. Data are presented as mean ± standard deviation and were analyzed by one-way ANOVA. * *p* < 0.05, *** *p* < 0.001, **** *p* < 0.0001 compared to the choline-deficient high-fat (CDHF) group. Obeticholic acid (OCA) was used as a positive control.

Based on Sirius red staining, the area of fibrosis significantly decreased due to the administration of ALS-L1023 (CDHF group [*n* = 10]: 11.4 ± 0.6%, OCA group [*n* = 9]: 9.3 ± 0.5%, ALS-L1023 low dose group [*n* = 10]: 7.0 ± 0.5% [*p* < 0.0001], ALS-L1023 high dose group [*n* = 7]: 7.4 ± 0.4% [*p* < 0.0001]; Figure 2A). Notably, the anti-fibrotic effect of ALS-L1023 appeared to be greater than that of OCA. However, evaluation of the H&E-stained liver tissue revealed that ALS-L1023 had no significant effect on NAS improvement (CDHF group: 7.6 ± 0.7, OCA group: 6.9 ± 0.8, ALS-L1023 low-dose group: 7.7 ± 0.6, ALS-L1023 high-dose group: 7.1 ± 0.9; Figure 2B).

### 3.3. ALS-L1023 Decreased the Expression of Fibrogenic Markers

**Figure 3:**
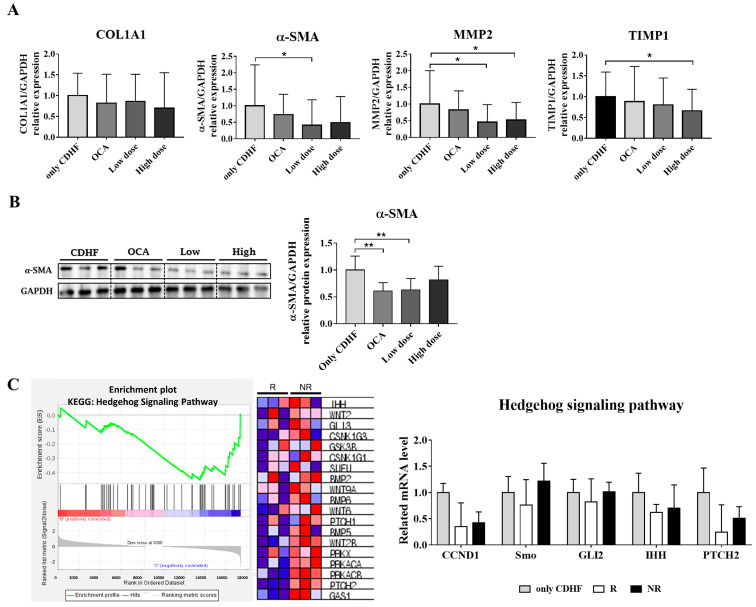
Effect of ALS-L1023 on the mRNA and protein expression of fibrosis markers, and the transcriptomic analysis for further verification. (**A**) mRNA expression of fibrosis markers in liver tissue. (**B**) protein expression of fibrosis marker in liver tissue. (**C**) Gene Set Enrichment Analysis and expression of related genes in the hedgehog-signaling pathway. Data are presented as mean ± standard deviation and were analyzed by Mann-Whitney U test. * *p* < 0.05, ** *p* < 0.01 compared to the choline-deficient high-fat (CDHF) group. Obeticholic acid (OCA) was used as a positive control. In the heat map, expression values are represented as the range of colors (red, pink, light blue, dark blue) that shows the range of expression values (high, moderate, low, lowest).

Based on the favorable anti-fibrotic effect observed in the histological analysis, we evaluated the expression of genes involved in liver fibrosis. The mRNA level of alpha-smooth muscle actin (α-SMA), a valuable marker in the reflection of stellate cell activation, was significantly decreased in the ALS-L1023 low-dose group compared to the CDHF group (Figure 3A, *p* = 0.035). Further, the high-dose group displayed a decreasing trend; however, the decrease was not significant. The expression level of TIMP metallopeptidase inhibitor 1 (TIMP1), which plays a key role in liver fibrosis associated with extracellular matrix composition, was significantly reduced in the ALS-L1023 high-dose group compared to the CDHF group, with a dose-dependent decreasing pattern observed (Figure 3A, *p* = 0.0306). In addition, matrix metalloproteinase-2 (MMP2), which is highly expressed in myofibroblasts and plays a profibrogenic role, was significantly decreased in both the ALS-L1023 low-and high-dose groups compared to the CDHF group (Figure 3A; CDHF group vs. ALS-L1023 low-dose group: *p* = 0.0160, CDHF group vs. ALS-L1023 high-dose group: *p* = 0.0386). Furthermore, gene expression of collagen 1A1 (COL1A1), a major component of type I collagen, showed a tendency to decrease in proportion to the dose of ALS-L1023. The protein expression of α-SMA was also significantly decreased in the ALS-L1023 low-dose group, which was comparable to that in the OCA group (Figure 3B; CDHF group vs. ALS-L1023 low-dose group: *p* = 0.003, CDHF group vs. OCA group: *p* = 0.0022).

### 3.4. ALS-L1023 May Exert Anti-Fibrotic Effects via the Hedgehog-Signaling Pathway

As fibrosis improvement was observed in the ALS-L1023 low-dose group, RNA sequencing was performed after mice were divided into responder and non-responder groups to explore the possible mechanism of the anti-fibrotic effect exhibited by ALS-L1023. We divided responders and non-responders based on the average area of fibrosis: classification as responders in the top 50% and non-responders in the bottom 50%. A comparison of the anti-fibrosis effect for responders and non-responders is provided in Supplementary Figure S1. We compared the responder and the non-responder groups based on the KEGG pathway using the GSEA tool and found that the hedgehog-signaling pathway-related genes were significantly reduced in the responder group (Figure 3C). When the expression of genes related to the hedgehog-signaling pathway was further examined, the responder group was found to have a lower expression of all related genes than the non-responder group, including Cyclin D1 (*Ccnd1*), Smoothened (*Smo*), GLI Family Zinc Finger 2 (*Gli2*), Indian Hedgehog (*Ihh*), and Patched 2 (*Ptch2*). These results suggest that ALS-L1023 may have an anti-fibrotic effect via a decrease in the hedgehog-signaling pathway, which needs further study to confirm.

### 3.5. ALS-L1023 Decreased the Expression of Hedgehog-Signaling Markers along with Anti-Angiogenic Effect

**Figure 4:**
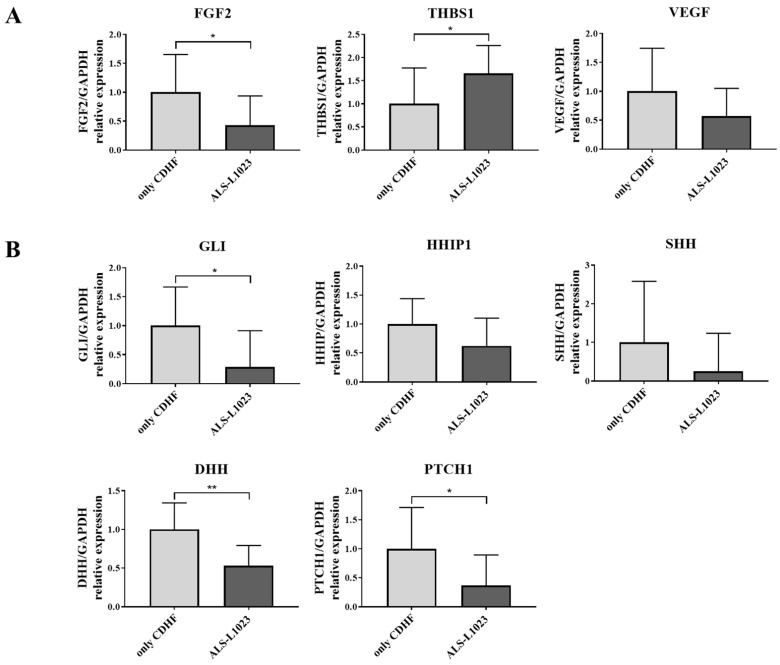
Effects of ALS-L1023 on the mRNA expression of anti-angiogenesis and hedgehog signaling pathway markers. (**A**) mRNA expression of angiogenic factors (VEGF and FGF2) and anti-angiogenic factor (THBS1) in liver tissue. (**B**) mRNA expression of genes related to the hedgehog signaling pathway. ALS-L1023 was treated with dose of 800 mg/kg/day. Data are presented as mean ± standard deviation and were analyzed by Mann-Whitney U test. * *p* < 0.05, ** *p* < 0.01 compared to the choline-deficient high-fat (CDHF) group.

To determine whether the hedgehog-signaling pathway is inhibited due to ALS-L1023, we investigated the expression profiles of other genes involved in the hedgehog-signaling pathway along with the anti-angiogenic effect. ALS-L1023 treatment decreased mRNA expression of angiogenic factors and increased expression of an anti-angiogenic factor in ALS-L1023-treated liver tissue. Both vascular endothelial growth factor (VEGF) and fibroblast growth factor 2 (FGF2) are strong activators of angiogenesis and thrombospondin1 (THBS1) is a potent anti-angiogenic factor which directly inhibits the angiogenic factors of endothelial cells. ALS-L1023 treatment decreased the mRNA levels of VEGF and FGF2, while it increased THBS1 expression (Figure 4A). In the meantime, ALS-L1023 treatment showed that the mRNA levels of genes regarding the hedgehog-signaling pathway including GLI1, HHIP1, SHH, DHH, and PTCH1 decreased compared with the CDHF diet alone (Figure 4B). These results suggest that ALS-L1023 can exhibit an anti-fibrosis effect via downregulation of the hedgehog-signaling pathway with an anti-angiogenic effect.

## 4. Discussion

We evaluated the efficacy of the anti-angiogenic lemon balm extract, ALS-L1023, in a CDHF-induced NAFLD model. Our results indicated that the administration of ALS-L1023 not only decreased ALT and AST levels but also alleviated liver fibrosis. RNA sequencing was also conducted to explore the mechanism of action of the anti-fibrotic effect exerted by ALS-L1023. Accordingly, the hedgehog-signaling pathway, which is a pathway that regulates the activation of hepatic stellate cells and promotes liver fibrosis, was found to be decreased by ALS-L1023 administration. These results suggest that the anti-fibrotic effect of ALS-L1023 correlates with the hedgehog-signaling pathway.

Since angiogenesis is a fundamental step in the formation of liver fibrosis, several anti-angiogenic compounds have been examined for potential antifibrotic efficacy. Sorafenib, one of the multi-kinase inhibitors as a first-line treatment for advanced hepatocellular carcinoma, exhibited anti-angiogenic and anti-fibrotic effects in preclinical studies [24,25,26]. Brivanib is a dual vascular endothelial growth factor receptor 2 (VEGFR-2)/fibroblastic growth factor receptor 1 (FGFR1) kinase inhibitor, which reduced hepatic fibrosis and activation of stellate cells through the inhibition of VEGF, FGF, and platelet-derived growth factor (PDGF) signaling in preclinical research [27,28,29]. In addition, L1-10 is a selective inhibitor of an angiogenic factor, angiopoietin-2 contributing to the progression of NASH, which decreased the inflammation, ballooning, and fibrosis in the liver with mice by inhibiting pathological vascular growth and endothelial cell dysfunction [30,31,32]. Other commonly used drugs including ezetimibe, losartan, and sitagliptin, or phytotherapeutic compounds with anti-angiogenic properties including berberine and ALS-L-1023 may be the potential therapeutic for NASH by inhibiting the angiogenic process [33].

Anti-angiogenic effect of ALS-L1023 has already been shown in previous research. ALS-L1023 showed anti-angiogenic and suppression of MMP activities, thereby reducing adipose tissue in obese mice [13,18]. In addition, the effect on angiogenesis for ALS-L1023 was confirmed that human umbilical vein endothelial cell-tube formation was inhibited in vitro, with the decreased formation of capillary-like tubular networks [15]. Further in vivo angiogenesis studies showed that ALS-L1023 impeded the vasculature of visceral adipose tissue [15]. ALS-L1023 treatment to obese mice reduced von Willebrand Factor (vWF)-positive cells and blood-vessel density, which was consistent with reduced mRNA expression of angiogenic factor VEGF-A while increasing mRNA expression of anti-angiogenic factor TSP-1 in visceral adipose tissue [15,16].

Liver fibrosis is a highly integrated consequence of a sustained wound-healing response that drives the progression of chronic liver diseases, which is characterized by excessive accumulation of extracellular matrix proteins [34]. In previous studies, the administration of ALS-L1023 caused a decrease in hepatic fibrosis in HFD-fed mice, which was supported by histological analysis and the decreased expression of fibrogenic genes [16] Furthermore, ALS-L1023 caused a decrease in pancreatic fibrosis in obese mice, possibly by modulating the gene expression responsible for fibrosis [35]. Similarly, our results indicate that ALS-L1023 could improve liver fibrosis in a CDHF-induced NAFLD model; however, research on the mechanism of anti-fibrosis is still limited.

Based on transcriptome analysis, the anti-fibrotic effect of ALS-L1023 may be due to a reduction in the gene set related to the hedgehog-signaling pathway. The hedgehog-signaling pathway is a conserved pathway, and its unrailed activation can result in malignancies, including medulloblastoma and basal cell carcinoma [36]. This pathway has also been reported to correlate with fibrosis caused by chronic liver injury [37]. Indian (IHH) is one of the three protein ligands that binds to its receptor, Patched (PTCH) [38], which reduces PTCH-regulated inhibition of Smoothened (Smo), thereby allowing the accumulation of Smo and activation of the major transcription factor, Gli2/3 [39,40,41]. In liver fibrosis, the hedgehog-signaling pathway contributes to the differentiation of quiescent hepatic stellate cells into proliferative myofibroblasts, which further produce extracellular matrix proteins, including collagen [42]. Previous studies revealed that the conditional deletion of SMO in myofibroblasts decreased fibrosis in the liver [43], and cyclopamine, a specific inhibitor of the hedgehog-signaling pathway via direct binding to Smo, reverted myofibroblastic transition by decreasing mesenchymal gene expression in rodent and human hepatic stellate cells [44,45]. In our GSEA based on KEGG, the administration of ALS-L1023 reduced the expression of genes related to the hedgehog-signaling pathway, including *Ihh*, *Gli2*, and *Smo*. Based on verification at the tissue RNA level, the related genes were found to be decreased in the responder group; however, the difference was not significant. Such findings must be confirmed by increasing the number of samples. Therefore, it is suggested that the anti-fibrotic effect of ALS-L1023, a lemon balm extract that serves as an angiogenesis inhibitor, is related to the regulation of the hedgehog-signaling pathway.

OCA is a first-in-class selective farnesoid X receptor (FXR) agonist that possesses anti-cholestatic and hepatoprotective effects [46] and is approved as Ocaliva for the treatment of primary biliary cholangitis [47]. Positively targeting FXR is a promising strategy for novel therapeutic options for NAFLD [48]. OCA acts as a critical regulator of bile acid metabolism and cholesterol lipoprotein, thereby modulating inflammatory and fibrogenic responses [47]. However, the undesirable effect of an atherogenic lipoprotein profile has recently become a challenging obstacle for drug approval, even though OCA significantly improves fibrosis, which may be of clinical benefit [49,50]. We used OCA as a positive control in our research; however, the administration of OCA resulted in marginal fibrosis reduction, with limited improvement in NAS. These controversial results were previously reported in several studies, where OCA was found to cause insufficient improvement either for fibrosis or steatosis in NAFLD [51,52] or potentially induce liver steatosis, ballooning, and inflammation [53]. On the other hand, the possible reason for the marginal effect of OCA in this study can be that the administered OCA dose of 10 mg/kg/day was a lower than other reported doses (10–30 mg/kg/day), but there are many cases in which 10 mg/kg of OCA is used [54,55,56,57,58,59,60].

Angiogenesis is critical in the etiology of cirrhosis and is associated with portal hypertension, underlying its progression and attributing to related complications [61]. Hepatic vascular resistance to portal blood flow is increased by newly created blood vessels that bypass sinusoids in liver cirrhosis and fail to give oxygen and nutrients to the tissues [61]. Anti-angiogenic therapy, which specifically inhibits the development and proliferation of newly created vessels, is a pathogenetically therapeutic approach for portal hypertension. Since most anti-angiogenic drug candidates were studied only in animals, ALS-L1023 could be the selective therapy for portal hypertension and its complications [62].

There are some limitations in this study. First, we did not investigate the anti-angiogenic effect in connection with the anti-fibrosis effect of ALS-L1023. It would have been better to supplement our analysis with data on hepatic angiogenesis, for instance by vascular corrosion casting, μCT scanning, and/or specific staining for blood vessels. Instead, we could refer to a previous study that confirmed the anti-angiogenic effect of ALS-L1023 by staining vWF in blood vessels [15], and based on this, we will be able to conduct a further study that can link angiogenesis and anti-fibrosis with hedgehog signaling. Second, although we present the anti-fibrotic effect of ALS-L1023 through this study, more reliable and sufficient data should be collected. In previous studies, α-SMA staining showed that α-SMA-positive cells were reduced by the administration of ALS-L1023, which was consistent with our results [15,16].

## 5. Conclusions

Our results reveal that the lemon balm extract, ALS-L1023, an angiogenesis inhibitor, might improve liver fibrosis via the hedgehog-signaling pathway in a CDHF-induced NAFLD model. ALS-L1023 may be a possible therapeutic compound for the treatment of liver fibrosis.

## Figures and Tables

**Table 1:** Primer sequences used for quantitative real-time polymerase chain reaction.

Primer	Sequence
Mouse GAPDH	F: 5′-GTT GTC TCC TGC GAC TTC-3′
	R: 5′-GGT GGTCCA GGG TTT CTT-3′
Mouse COL1A1	F: 5′-CTG GAC AAC GTG GTG TGG-3′
	R: 5′-TGT TTG CCA GGT TCA CCA-3′
Mouse α-SMA	F: 5′-TCG GAT ACT TCA GCG TCA GGA-3′
	R: 5′-GTC CCA GAC ATC AGG GAG TAA-3′
Mouse MMP2	F: 5′-GCT GTA TTC CCG ACC GTT GA-3′
	R: 5′-TGG TCC GCG TAA AGT ATG GG-3′
Mouse TIMP1	F: 5′-ATC TCT GGC ATC TGG CAT CC-3′
	R: 5′-TTG CAG AAG GCT GTC TGT GG-3′
Mouse CCND1	F: 5′-CGC CCT CCG TAT CTT ACT TCA-3′
	R: 5′-TCT TCG CAC TTC TGC TCC TC-3′
Mouse Smo	F: 5′-GCG GGT TTG GCT TTT GAC-3′
	R: 5′-GGA CAC ATC CTG GGG CAA TA-3′
Mouse GLI1	F: 5′-GCC TTG AAA ACC TCA AGA CG-3′
	R: 5′-ATG GCT TCT CAT TGG AGT GG-3′
Mouse GLI2	F: 5′-AGT GGA ATG AGG TGA GTT CTG G-3′
	R: 5′-AGG CTG GCT TCT GTT GGA C-3′
Mouse HHIP1	F: 5′-GAA GGA GAT GCG AAG TTT GG-3′
	R: 5′-CCC TTC TCT TTA GGC GCT TT-3′
Mouse IHH	F: 5′-TCA AGG ACG AGG AGA ACA CG-3′
	R: 5′-ACC CGC AGT TTC ACA CCA-3′
Mouse SHH	F: 5′-CTG GTG ATC CTT GCT TCC TC-3′
	R: 5′-GGC TAA AGG GGT CAG CTT TT-3′
Mouse DHH	F: 5′-CAA GCA GTT TGT GCC CAG TA-3′
	R: 5′-GTC GGG GTT GTA GTT GGG TA-3′
Mouse PTCH1	F: 5′-GGA GCT CAG GCA ATA CGA AG-3′
	R: 5′-GGA GGC TGA TGT CTG GAG TC-3′
Mouse PTCH2	F: 5′-GCC TGC GTA ACA ATG CTG-3′
	R: 5′- GCA CAA AGC CCA AGA CCT -3′
Mouse VEGF	F: 5′-GTA CCT CCA CCA TGC CAA GT-3′
	R: 5′-TCA CAT CTG CAA GTA CGT TCG-3′
Mouse FGF2	F: 5′-AGC GGC TCT ACT GCA AGA AC-3′
	R: 5′-GCC GTC CAT CTT CCT TCA TA-3′
Mouse THBS1	F: 5′-CCA AAG CCT GCA AGA AAG AC-3′
	R: 5′-CCT GCT TGT TGC AAA CTT GA-3′

GAPDH, Glyceraldehyde-3-Phosphate Dehydrogenase; COL1A1, Collagen Type I Alpha 1 Chain; α-SMA, α-smooth muscle actin; MMP2, Matrix Metallopeptidase 2; TIMP1, Tissue inhibitor matrix metalloproteinase 1; CCND1, Cyclin D1; Smo, smoothened; GLI, GLI Family Zinc Finger; HHIP1, hedgehog-interacting protein 1; IHH, Indian Hedgehog Signaling Molecule; SHH, Sonic Hedgehog Signaling Molecule; DHH, Desert Hedgehog Signaling Molecule; PTCH, Patched; VEGF, Vascular endothelial growth factor; FGF2, Fibroblast Growth Factor 2; THBS1, Thrombospondin-1.

## Data Availability

The data that support the findings of this study are available from the corresponding author, D.W.J., upon reasonable request.

## Supplementary Materials

The following supporting information can be downloaded at: https://www.mdpi.com/article/10.3390/life13010100/s1, Figure S1: Comparison of anti-fibrosis effect for responders and non-responders in the low dose group of ALS-L1023.
