# Supplementary material for: ALS-L1023 from Melissa officinalis Alleviates Liver Fibrosis in a Non-Alcoholic Fatty Liver Disease Model
Source: Life (Basel). 2022 Dec 29;13(1):100. doi: 10.3390/life13010100 (PMC9863634; doi:10.3390/life13010100)
Supplement: Supplementary file 1 [file life-13-00100-s001.zip › life-2059981-supplementary.pdf]

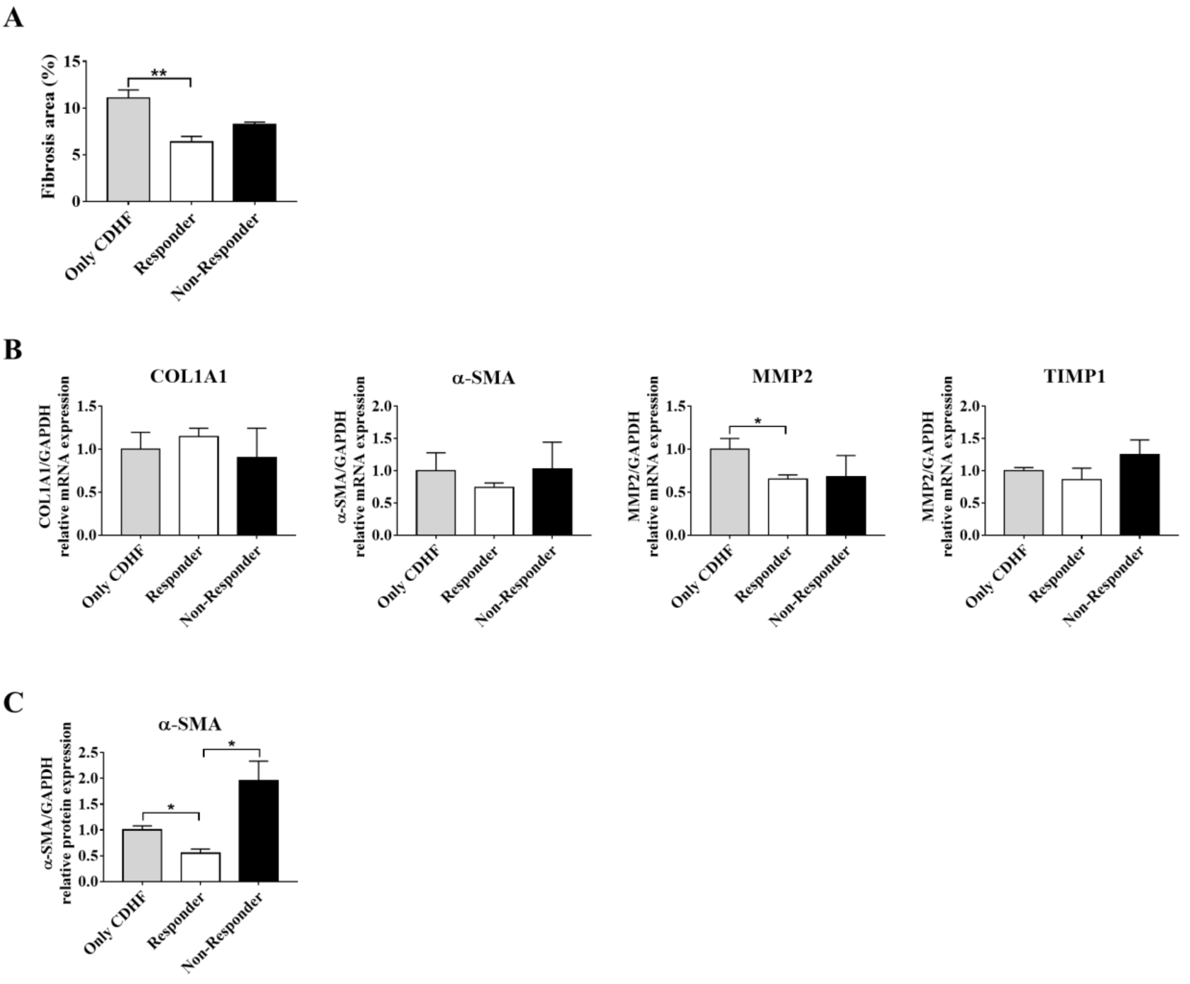

Supplementary Figure S1. Comparison of anti-fibrosis effect for responders and non-responders in the low dose group of ALS-L1023.

(**A**) Area of fibrosis. (**B**) mRNA expression of fibrosis markers in liver tissue. (**C**) protein expression of fibrosis marker in liver tissue. Data are presented as mean ± SEM, analyzed by t-test and one-way ANOVA. *$p<0.05$ and **$p<0.01$ for statistical significance.
